# A Long-Term Enriched Environment Ameliorates the Accelerated Age-Related Memory Impairment Induced by Gestational Administration of Lipopolysaccharide: Role of Plastic Mitochondrial Quality Control

**DOI:** 10.3389/fncel.2020.559182

**Published:** 2021-02-03

**Authors:** Zhan-Qiang Zhuang, Zhe-Zhe Zhang, Yue-Ming Zhang, He-Hua Ge, Shi-Yu Sun, Ping Zhang, Gui-Hai Chen

**Affiliations:** ^1^Department of Neurology (Sleep Disorders), The Affiliated Chaohu Hospital of Anhui Medical University, Hefei, China; ^2^Division of Life Sciences and Medicine, Department of Neurology, The First Affiliated Hospital of USTC, University of Science and Technology of China, Hefei, China; ^3^Institute of Sleep Medicine of Anhui Medical University, Hefei, China

**Keywords:** enriched environment, lipopolysaccharide, age-related memory impairment, mitochondrial quality control, hippocampus

## Abstract

Studies have shown that gestational inflammation accelerates age-related memory impairment in mother mice. An enriched environment (EE) can improve age-related memory impairment, whereas mitochondrial dysfunction has been implicated in the pathogenesis of brain aging. However, it is unclear whether an EE can counteract the accelerated age-related memory impairment induced by gestational inflammation and whether this process is associated with the disruption of mitochondrial quality control (MQC) processes. In this study, CD-1 mice received daily intraperitoneal injections of lipopolysaccharide (LPS, 50 μg/kg) or normal saline (CON group) during gestational days 15–17 and were separated from their offspring at the end of normal lactation. The mothers that received LPS were divided into LPS group and LPS plus EE (LPS-E) treatment groups based on whether the mice were exposed to an EE until the end of the experiment. At 6 and 18 months of age, the Morris water maze test was used to evaluate spatial learning and memory abilities. Quantitative reverse transcription polymerase chain reaction and Western blot were used to measure the messenber RNA (mRNA) and protein levels of MQC-related genes in the hippocampus, respectively. The results showed that all the aged (18 months old) mice underwent a striking decline in spatial learning and memory performances and decreased mRNA/protein levels related to mitochondrial dynamics (Mfn1/Mfn2, OPA1, and Drp1), biogenesis (PGC-1α), and mitophagy (PINK1/parkin) in the hippocampi compared with the young (6 months old) mice. LPS treatment exacerbated the decline in age-related spatial learning and memory and enhanced the reduction in the mRNA and protein levels of MQC-related genes but increased the levels of PGC-1α in young mice. Exposure to an EE could alleviate the accelerated decline in age-related spatial learning and memory abilities and the accelerated changes in MQC-related mRNA or protein levels resulting from LPS treatment, especially in aged mice. In conclusion, long-term exposure to an EE can counteract the accelerated age-related spatial cognition impairment modulated by MQC in CD-1 mother mice that experience inflammation during pregnancy.

## Introduction

An expanding elderly population globally has led to an increase in age-related disorders, such as Alzheimer's disease (AD). The consequently increased rates of cognitive dysfunction impose increasing societal, economic, and medical burdens (Michalowsky et al., 2019). However, the precise pathogenesis associated with this cognitive impairment remains elusive, and no ideal treatment currently exists (Perneczky, 2019). Hence, there is an urgent need to understand the underlying pathogenesis and explore effective treatments to reduce the adverse impact of age-related cognitive decline.

Besides supplying cellular energy, mitochondria are also indispensable for lipid metabolism (Oleinik et al., 2019), the synthesis of amino acids (Corbet and Feron, 2017), Ca^2+^ homeostasis (Zavodnik, 2016), iron/sulfur cluster biosynthesis (Wachnowsky et al., 2018), regulation of apoptosis, and other signaling processes (Kaczanowski, 2016). This highlights the importance of mitochondria in maintaining cellular and human health. According to the mitochondrial theory, aging results from accumulated mitochondrial damage (Kong et al., 2014). Increasing evidence has indicated that mitochondrial dysfunction can contribute to the pathophysiology of age-related disorders, including neurodegenerative diseases, diabetes, cardiovascular diseases, cancers, and sarcopenia (Lane et al., 2015; Bhatti et al., 2017; Elfawy and Das, 2019). Indeed, therapeutic approaches targeting mitochondria in age-related diseases are currently a very promising research path.

Mitochondrial dysfunction and chronic inflammation are closely associated with the aging process (Dodig et al., 2019). On the one hand, mitochondria are the main producers of reactive oxygen species (ROS), which serve as major proinflammatory stimulants. ROS-induced injury accumulation can lead to mitochondrial dysfunction (Picca et al., 2017). Furthermore, ROS can activate target of rapamycin, which can, in turn, lead to increased ROS production. Activated target of rapamycin signaling can promote the accumulation of lipofuscin, defective mitochondria, and aggregation-prone proteins, resulting in aging (Blagosklonny, 2008). On the other hand, circulating cell-free mitochondrial DNA, an important cell damage-associated molecular pattern, can induce a proinflammatory cascade through nuclear factor kappa B signaling and the NOD-like receptor protein 3 inflammasome (Picca et al., 2017). They are related to one another and trigger aging and age-related neurodegeneration (Mathew et al., 2012; Dodig et al., 2019). Lipopolysaccharide (LPS), widely used in animal experiments to simulate bacterial infection-related diseases, can trigger severe systemic inflammation and lead to mitochondrial dysfunction and neuronal damage (Hunter et al., 2007). We have previously shown that exposure to LPS during pregnancy can significantly accelerate age-related cognitive impairment and changes in hippocampal neurobiochemical processes in female CD-1 mice (Li et al., 2016). These effects in middle-aged (15 months old) CD-1 female mice resulting from LPS-induced gestational inflammation can be attributable, at least in part, to enhanced mitochondrial dysfunction at this age (Noh et al., 2014). In response to stress, mitochondria activate several quality control (MQC) mechanisms, including mitochondrial dynamics, biogenesis, and mitophagy, which play an important role in ROS scavenging, repair and refolding of damaged mitochondrial proteins, and removal and replacement of irreversibly damaged mitochondria (Fischer et al., 2012). These processes are coordinated to ensure mitochondrial homeostasis, thereby promoting cell survival and organ function.

Mitochondria are dynamic, double-membrane bound organelles that undergo continuous cycles of fission and fusion, known as mitochondrial dynamics. Mitofusin-1/2 (Mfn1/2), optic atrophy 1 (OPA1), and dynamin-related protein 1 (Drp1) are key regulators of mitochondrial dynamics (Ni et al., 2015). Mfn1/2 and OPA1 are involved in the fusion of the outer and inner mitochondrial membranes, whereas Drp1 is a major player in mitochondrial fission. Fusion is a content-mixing process involving both depolarized and healthy mitochondria, which enables them to share mitochondrial membrane potential, as well as protein and DNA contents (Twig et al., 2008; Ni et al., 2015). Hence, fusion can compensate for mitochondrial defects. Fission divides damaged mitochondria into two asymmetrical daughter mitochondria, one of which has a normal membrane potential and can fuse with other mitochondria (Twig et al., 2008). However, the other daughter mitochondrion has a reduced membrane potential, which prevents fusion with other healthy mitochondria and results in the elimination of defective organelles (Twig et al., 2008). Therefore, mitochondrial dynamics are a crucial MQC mechanism. Numerous studies have shown that the levels of Drp1 are increased, whereas those of Mfn1, Mfn2, and OPA1 are decreased, in mouse models of AD (Kandimalla et al., 2018); the accumulation of mutant amyloid precursor protein and beta-amyloid peptide is responsible for these dysregulated mitochondrial dynamics (Reddy et al., 2018).

Mitochondrial biogenesis is a well-orchestrated process that replenishes these organelles with newly synthesized membrane lipids, respiratory chain components, and enzymes. Peroxisome proliferator-activated receptor-γ coactivator 1-alpha (PGC-1α) is a major regulator of mitochondrial biogenesis (Gleyzer et al., 2005) and can promote the expression of mitochondrial transcription factor A/B (TFAM/TFBM), a regulator of mitochondrial gene transcription (Gleyzer et al., 2005). When encountering external or internal stimuli, including exercise or inflammation, PGC-1α is involved in the regulation of mitochondrial biogenesis to meet cellular requirements (Silva S. C. A et al., 2019). PGC-1α can be considered an antiaging factor, as its levels exhibit an age-related decline and also because it can be induced by mutant amyloid precursor protein and beta-amyloid peptide accumulation in the hippocampus (Wenz, 2011).

Mitochondrial autophagy (mitophagy) is a type of selective autophagy that leads to the selective removal of impaired mitochondria and maintains a healthy mitochondrial population (Leites and Morais, 2018). The PTEN-induced putative kinase 1 (PINK1)/parkin signaling pathway is a key mediator of mitophagy (Leites and Morais, 2018). When mitochondria become depolarized, PINK1 translocates from the inner to the outer mitochondrial membrane and then phosphorylates and recruits cytosolic parkin to mitochondria in itself a phosphorylation-dependent manner. Activated parkin ubiquitinates outer-membrane proteins of depolarized mitochondria (McWilliams and Muqit, 2017). The ubiquitinated mitochondrial proteins directly or indirectly interact with autophagic proteins and promote their proteasome-dependent degradation of these ubiquitinated proteins (McWilliams and Muqit, 2017). Numerous studies have indicated that mitophagy efficiency decreases with age, resulting in the accumulation of impaired mitochondria and deterioration of cell function in older individuals (Shi et al., 2018). Age-dependent increased levels of beta-amyloid and hyperphosphorylated tau significantly reduce the levels of several mitophagy-related proteins in AD (Reddy and Oliver, 2019). This defective mitophagy contributes to the progressive pathology of age-related diseases. Hence, modulation of mitophagy represents a potentially new therapeutic target for the treatment of age-associated pathologies.

Mounting evidence suggests that mitochondrial dysfunction is a hallmark of age-related pathologies (Lane et al., 2015). The earlier mentioned MQC mechanisms are integrated to maintain mitochondrial network homeostasis (Fischer et al., 2012). Modulation of MQC has potential as a novel therapeutic intervention to counteract age-associated pathological conditions (Picca et al., 2018). Both the use of natural compounds or drugs, such as spermidine and rapamycin, and lifestyle interventions, such as calorie restriction and physical exercise, can offset the deterioration of mitochondrial function and prevent inflammation, metabolic disorders, neurodegeneration, and loss of homeostasis during aging through their influence on MQC processes (Fischer et al., 2012; Suliman and Piantadosi, 2016). These observations indicate that MQC is an important target in aging research.

Brain aging is a multifactorial process. Poverty, infection, low education level, and adverse lifestyle are key risk factors for this process (Reitz and Mayeux, 2014). Pregnancy affects metabolic processes and homeostasis. The physiological and cellular degradation seen in pregnancy mimic similar mechanisms associated with aging (Giller et al., 2020). Hence, it provides a unique platform for studying aging. LPS-induced memory impairment can be regarded as model for AD (Zakaria et al., 2017). Pregnant animals have increased sensitivity to LPS. So, LPS treatment during pregnancy can better simulate the clinical scenario of accelerated brain aging. whereas positive stress has a protective effect on brain aging (Dumas, 2017). Exposure to an enriched environment (EE), which is a combination of enlarged physical space, larger group size, and the presence of running wheels and other objects used for exercise and hiding, is a classical model used in neuroscience for studying eustress impacts (Nithianantharajah and Hannan, 2006). EEs can promote hippocampal neurogenesis, induce neural plasticity and mitochondrial activity, and improve cognitive function (Nithianantharajah and Hannan, 2006). Studies have indicated that an EE can alleviate the deficits in the learning and memory capabilities of aged mice (Cintoli et al., 2018). However, how an EE affects the accelerated age-related cognitive impairment resulting from gestational exposure to LPS, and the likely accompanying changes in MQC processes, remains unknown.

Therefore, in this study, we first explored whether accelerated memory impairment in aged CD-1 pregnant mice exposed to LPS during pregnancy could be ameliorated by exposure to an EE. Secondly, we investigated whether gestational exposure to LPS worsened age-related changes in MQC-related mRNA or protein expression, and whether this deterioration could be ameliorated by an EE. Finally, we examined the correlations between age-related memory impairment and changes in the mRNA or protein expression of MQC-related genes.

## Materials and Methods

### Animals and Experimental Procedures

**Figure 1:**
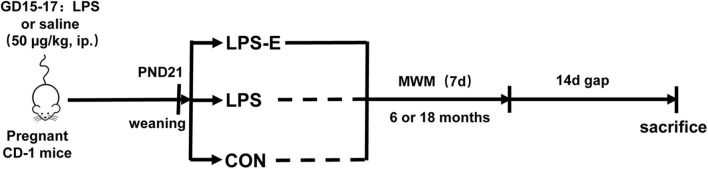
Timeline of experimental events. Pregnant mice were intraperitoneally injected with LPS or normal saline on days 15–17 of gestation (GD). Their offspring were weaned at post-natal day (PND) 21, and this mothers were divided into three groups based on whether the mice were exposed to an EE until the end of the experiment. MWM test was performed at 6 and 18 months of age. Fourteen days after the MWM test, mice were killed for subsequent biochemical experiments. CON, control group; LPS, lipopolysaccharide treatment group; LPS-E, Lipopolysaccharide plus enriched environment treatment group; MWM, Morris water maze.

All animal-related procedures and animal care were in accordance with the approved Institutional Animal Care and Use Protocols of Anhui Medical University, and this study was approved by the Animal Ethics Committee of the University. The CD-1 mice (7–8 weeks old, 45 males and 90 females) were purchased from the Model Animal Research Center of Nanjing University. All the mice were housed in standard plastic mouse cages (25.5 × 15 × 14 cm^3^) under controlled, standard laboratory conditions (room temperature: 24 ± 2°C; humidity: 55 ± 10%; 12-h light/dark cycle with lights on at 07:00; food and water supplied *ad libitum*). The animals were acclimated to the laboratory conditions 2 weeks before the experiment. Males and females were mated (1:2) overnight immediately for breeding after the adaptation. The next morning, the presence of a vaginal plug was regarded as gestational day 0. During gestational days 15–17, all the pregnant females received a daily intraperitoneal injection of LPS (50 μg/kg) or normal saline [control (CON) group] (Li et al., 2016). At the end of normal lactation, the mothers were separated from their offspring at post-natal day 21 and maintained in standard home cages (three to four mice per cage). Half the mice that had been treated with LPS were placed in enlarged cages (52 × 40 × 20 cm^3^, 10–15 mice per cage) containing a varied assortment of toys, such as running wheels, tunnels, poplar wood block toys, and rings, to provide an EE until the end of the behavioral experiments (defined as the LPS-E group). When the mother mice had reached 6 and 18 months of age, ten mice were randomly selected from each of the three groups to complete the subsequent experiments. The schematic representation of the experimental timeline is shown in Figure 1.

### Morris Water Maze Test

The Morris water maze (MWM) test was adopted to assess the hippocampal-dependent spatial learning and memory abilities of the mice. The protocol used in this study was as previously reported (Tong et al., 2015). The experimental apparatus consisted of a circular black pool (diameter 120 cm, height 30 cm) filled with clear water (22–24°C), containing a mobile black escape platform (diameter 10 cm, height 24 cm). The periphery of the pool was surrounded by a white curtain from the ceiling to the ground containing three black cardboard shapes (square, triangle, and circle) 1.5 m from the bottom of the pool that served as spatial cues. The pool was divided into four quadrants. First, all the mice were trained to find a visible escape platform 1 cm above the water surface for five times to determine whether they had good vision. In the visible platform task (without the surrounding curtain), a flag was placed on the platform in continually varying positions, whereas the starting point of the mice remained constant. The mice were given 60 s to find the escape platform. Regardless of whether or not they found the platform, they were made to remain on the platform for 30 s (the recorded results were not included in the statistical analyses). Subsequently, the mice underwent seven consecutive days of learning-ability testing (place navigation task). During this phase, the platform was submerged 1 cm below the water surface and fixed at the center of the target quadrant; the starting point was changed constantly among the four quadrants of the pool. Four learning trials were conducted every day with 15-min intervals between trials. The mice were required to find the platform within 60 s and allowed to remain on the platform for 30 s in each trial. The latency, distance swam, and mean velocity until reaching the platform were used to evaluate their spatial learning ability. On the last day, the platform was removed before the probe trial task was performed. Two hours after the last learning trial, the mice were placed in the quadrant opposite the platform position and allowed to swim for 60 s. The percent distance swam and the percent time spent swimming (the ratio of target quadrant distance or time to total swimming distance or time) in the probe trial task were used to evaluate memory retention. After the completion of each trial, the mice were dried using an electric heater. All the trials were completed in the dark period, and the behavioral parameters were recorded and analyzed by behavioral analysis software (Any-Maze, USA).

### Tissue Preparation

To avoid the possible influence of experimental manipulations on mRNA or protein expression, the mice were decapitated 2 weeks after the behavioral tests. The brains were promptly removed and bisected along the mid-sagittal suture on ice. The hippocampi were then rapidly isolated and frozen at −80°C. The right hippocampus was used for Western blotting and the left for quantitative real-time RT-PCR.

### Quantitative Real-Time Polymerase Chain Reaction

Total RNA was extracted from the left hippocampus using Trizol reagent following the manufacturer's instructions. The purity and content of the extracted RNA were assessed using a spectrophotometer. RNA (1 μg) was reverse-transcribed to complementary DNA (cDNA) using The RevertAid^TM^ First-Strand cDNA Synthesis Kit. The transcripts were amplified by quantitative real-time PCR using Novostart SYBR qPCR SuperMix Plus in a 10-μl reaction mixture containing 5 μl of 2 × SYBR Green mixture, 1 μl of each primer (10 μM), 1 μl of cDNA template, and 2 μl of RNase-free water. The quantitative real-time PCR reaction condition included one cycle of 95°C for 1 min and 40 cycles of 95°C for 20 s and 60°C for 1 min. The mRNA level was quantified using the 2^ΔΔ*Ct*^ method. Beta-actin served as the internal reference. The primer sequences are listed in Table 1.

**Table 1:** Sequences of the primers used for quantitative real-time PCR.

**Target gene**	**Forward primer (5^**′**^-3^**′**^)**	**Reverse primer (5^**′**^-3^**′**^)**
β-actin	AGTGTGACGTTGACATCCGT	TGCTAGGAGCCAGAGCAGTA
Pgc-1α	ACAACGCGGACAGAATTGAG	GTTTCGTTCGACCTGCGTAA
Mfn1	CCACAAGCTGTGTTCGGATT	CTGTGCATTTGTGGAACCCA
Mfn2	ACGGAGAAGCAGTTCTTCCA	CAGCTCATCCACCAGAAAGC
Drp1	GCAACTGGAGAGGAATGCTG	TGGAACTGGCACATCTAGCA
Opa1	AAGAATCGGACCCAAGAGCA	CTTCCACTCCTCGAGACTCC
Pink1	CCTGGCTACCATGATGACCT	AGTCCCACTCCACAAGGATG
Parkin	GCCTCATCTCCACTGAACCT	TACAGTCAATGCTGCCGTTG

### Western Blotting

Right hippocampal tissues were homogenized in radioimmunoprecipitation assay lysis buffer. The homogenates were centrifuged at 12,000 rpm for 10 min, and the supernatants were collected. The supernatants and 5 × sodium dodecyl sulfate loading buffer were mixed and boiled for 10 min. The target proteins were isolated using 10 or 15% sodium dodecyl sulfate–polyacrylamide gel electrophoresis and transferred onto polyvinylidene fluoride membranes by electrophoresis. Non-specific binding was blocked by incubation in 5% skim milk. The membranes were then incubated with anti-PGC-1α (1:200; SC-517353; Santa Cruz), anti-PINK1 (1:200; SC-517353; Santa Cruz), anti-OPA1 (1:200; SC-393296; Santa Cruz), anti-Mfn1 (1:2,000; 13798-1-AP; Proteintech), anti-Mfn2 (1:1,000; 12186-1-AP; Proteintech), anti-Drp1 (1:2,000; 12957-1-AP; Proteintech), anti-parkin (1:1,000; 14060-1-AP; Proteintech), and anti-β-actin (1:1,000; TA-09; ZS-BIO) antibodies overnight at 4°C. After washing three times with Tris-buffered saline with 0.1% Tween, the membranes were incubated with horseradish peroxidase-conjugated goat anti-mouse or anti-rabbit secondary antibodies (1:5,000; ZB-2305 or ZB-2301; ZS-BIO) for 2 h at room temperature and then washed three times again. The immunoreactive target protein bands were visualized using an enhanced chemiluminescence kit (Thermo, USA). The gray values of the protein bands were analyzed in ImageJ. Duplicate samples were averaged for each subject. Beta-actin served as the internal reference. To control for equal loading, the ratio of the optical density of the antibody of interest to that of the anti-β-actin antibody was calculated for each sample.

### Statistical Analysis

Normally distributed data (behavioral results, mRNA and protein results) were reported as means ± standard error of the mean (SEM). Repeated-measures analysis of variance was used to analyze the data from the MWM learning task, with day, treatment, or age as the independent variable. To determine the main effects of treatment, normally distributed data were analyzed using one-way analysis of variance with Fisher's least-significant difference *post hoc* test to compare the differences among the different groups at each age. The student's *t*-test was used to analyze the effect of age. The correlations between the MWM performance and the target mRNA or protein expression levels were analyzed using Pearson's correlation coefficient. Differences were considered significant at *P* < 0.05. All data analyses were performed using SPSS® 23.0 for Windows.

## Results

### Performance in the Morris Water Maze

#### Age Effects

**Figure 2:**
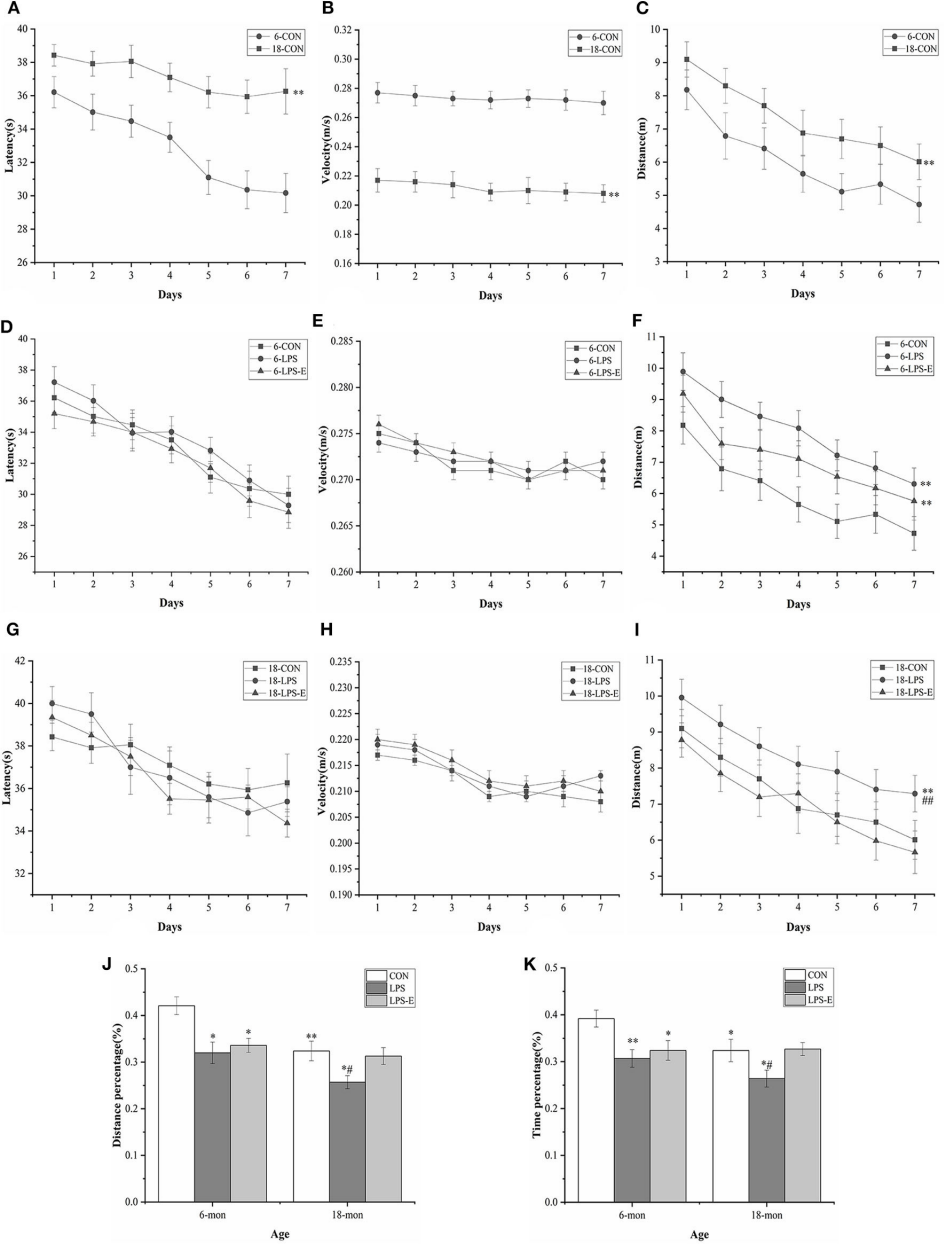
Performances in the Morris water maze (MWM) test. Latency **(A,D,G)**, average velocity **(B,E,H)**, and distance **(C,F,I)** during the learning phase (trials 1–4); percent distance swam **(J)** and percent time spent swimming **(K)** during the memory phase (trial 5). Age and treatment had a significant effect on learning and memory performance in the MWM. Data are expressed as means ± SEM. *Significant differences compared with 6- or 18-month-old CON mice (*P < 0.05, **P < 0.01); ^#^Significant differences compared with 6- or 18-month-old LPS-E mice (^#^*P* < 0.05, ^##^*P* < 0.01). 6: 6-month-old; 18: 18-month-old; CON, control group; LPS, lipopolysaccharide treatment group; LPS-E, LPS plus enriched environment treatment group.

In the learning phase, the swimming latency [*F*_(6,114)_ = 8.579, *P* < 0.001] and distance swam [*F*_(6,114)_ = 9.193, *P* < 0.001] gradually declined with an increase in the number of training days for all the control mice combined; the average velocity [*F*_(6,114)_ = 0.841, *P* = 0.541] decreased over time, although not significantly. Moreover, age had a significant effect on swimming latency [*F*_(1,18)_ = 52.311, *P* < 0.001], distance swam [*F*_(1,18)_ = 9.193, *P* < 0.001], and average velocity [*F*_(1,18)_ = 99.938, *P* < 0.001] in mice from the CON group (Figures 2A–C). However, the 18-month-old mice exhibited significantly greater swimming latency and distance swam, but slower average velocity, compared with the 6-month-old mice. The effect of interaction of group × day was not significant in the learning phase (*P*s > 0.05).

In the memory phase, the percent distance swam (*t* = 3.421, *P* = 0.003) and percent time spent swimming (*t* = 2.393, *P* = 0.028) within the target quadrant was significantly lower in the 18-month-old CON mice than in the 6-month-old CON mice (Figures 2J,K).

#### Treatment Effects

##### Six-Month-Old Mice

In the learning phase, the swimming latency [*F*_(6,162)_ = 31.174, *P* < 0.001] and distance swam [*F*_(6,162)_ = 11.576, *P* < 0.001] showed a gradual, daily decline in the different treatment groups; the average velocity also declined, although not significantly [*F*_(6,162)_ = 1.147, *P* = 0.247]. The effect of treatment was significant for the distance swam [*F*_(2,27)_ = 10.024, *P* = 0.001] but not for swimming latency [*F*_(2,27)_ = 0.045, *P* = 0.956] or average velocity [*F*_(2,27)_ = 1.819, *P* = 0.181] (Figures 2D–F). Mice in the LPS and LPS-E treatment groups had similar swimming latency and average velocity (*P* = 0.058); however, mice in both groups swam significantly longer distances than those in the CON group (*Ps* < 0.05, Figure 2F). The effect of interaction of group × day on distance swam was not significant (*Ps* > 0.05).

In the memory phase, the percent distance swam [*F*_(2,27)_ = 6.387, *P* = 0.005] and percent time spent swimming [*F*_(2,27)_ = 5.367, *P* = 0.011] within the target quadrant were significantly different among the groups. Furthermore, the percent distance swam and time spent swimming in the target quadrant were significantly lower in the LPS (*P* = 0.03 and 0.005) and LPS-E (*P* = 0.09 and 0.019) treatment groups than in the CON group (Figures 2J,K).

##### Eighteen-Month-Old Mice

Similarly, the learning latency [*F*_(6,162)_ = 9.097, *P* < 0.001] and distance swam [*F*_(6,162)_ = 12.275, *P* < 0.001], but not the average velocity [*F*_(6,162)_ = 0.985, *P* = 0.423], showed a progressive, daily decline for all the groups combined. The distance swam [*F*_(2,27)_ = 6.796, *P* = 0.004], but not the latency [*F*_(2,27)_ = 0.473, *P* = 0.628] or average velocity [*F*_(2,27)_ = 0.146, *P* = 0.865], differed significantly among groups (Figures 2G–I). A pairwise comparison indicated that LPS-treated mice swam significantly longer distances than mice in the LPS-E and CON groups (*P* = 0.002 and 0.010); no difference was observed between the LPS-E and CON groups (*P* = 0.423, Figure 2I). The effect of interaction of group × day on the learning parameters was not significant (*Ps* > 0.05).

Additionally, the percent distance swam [*F*_(2,27)_ = 4.274, *P* = 0.024] and percent time spent swimming [*F*_(2,27)_ = 3.391, *P* = 0.049] differed significantly among the groups; the percent distance swam (*P* = 0.011 and 0.030) and percent time spent swimming (*P* = 0.036 and 0.029) were both significantly smaller in the LPS treatment group than in the LPS-E and CON groups but were similar between the LPS-E and CON groups (Figures 2J,K).

### Dynamics of Hippocampal Mitochondria

#### Messenger RNA Levels of *Drp1, Mfn1, Mfn2*, and *Opa1*

As shown in Figures 4A–D, the mRNA levels of *Drp1* (*t* = 8.397, *P* < 0.001), *Mfn1* (*t* = 5.164, *P* = 0.003), *Mfn2* (*t* = 9.972, *P* < 0.001), and *Opa1* (*t* = 6.906, *P* = 0.001) were significantly reduced in the 18-month-old CON mice compared with the 6-month-old mice from the same treatment group. At 6 months of age, the hippocampal mRNA levels of *Drp1* [*F*_(2,15)_ = 10.317, *P* = 0.002], *Mfn2* [*F*_(2,15)_ = 24.474, *P* < 0.001], and *Opa1* [*F*_(2,15)_ = 10.684, *P* = 0.001], but not that of *Mf n1* [*F*_(2,15)_ = 2.559, *P* = 0.111], differed significantly among treatment groups. A pairwise comparison showed that mice in the LPS and LPS-E groups had significantly lower mRNA levels of *Drp1, Mfn2*, and *Opa1* than mice in the CON group (*Ps* < 0.001); mice in the LPS and LPS-E treatment groups exhibited similar levels of *Mfn1* mRNA (*Ps* > 0.596). At 18 months of age, the mRNA levels of all four genes differed among all the groups (Figures 4A–D) [*F*_(2,15)_ = 26.327, *P* < 0.001 for *Drp1*; *F*_(2,15)_ = 9.073, *P* = 0.003 for *Mfn1*; *F*_(2,15)_ = 20.324, *P* < 0.001 for *Mfn2*; and *F*_(2,15)_ = 20.054, *P* < 0.001 for *Opa1*]. Furthermore, the levels of all four mRNAs were significantly reduced in the LPS group relative to both the LPS-E group (*Ps* < 0.05) and the CON group (*Ps* < 0.001); only *Mfn2* presented lower mRNA levels in the LPS-E group compared with the CON group (*P* < 0.001).

#### Drp1, Mfn1, Mfn2, and OPA1 Protein Levels

**Figure 3:**
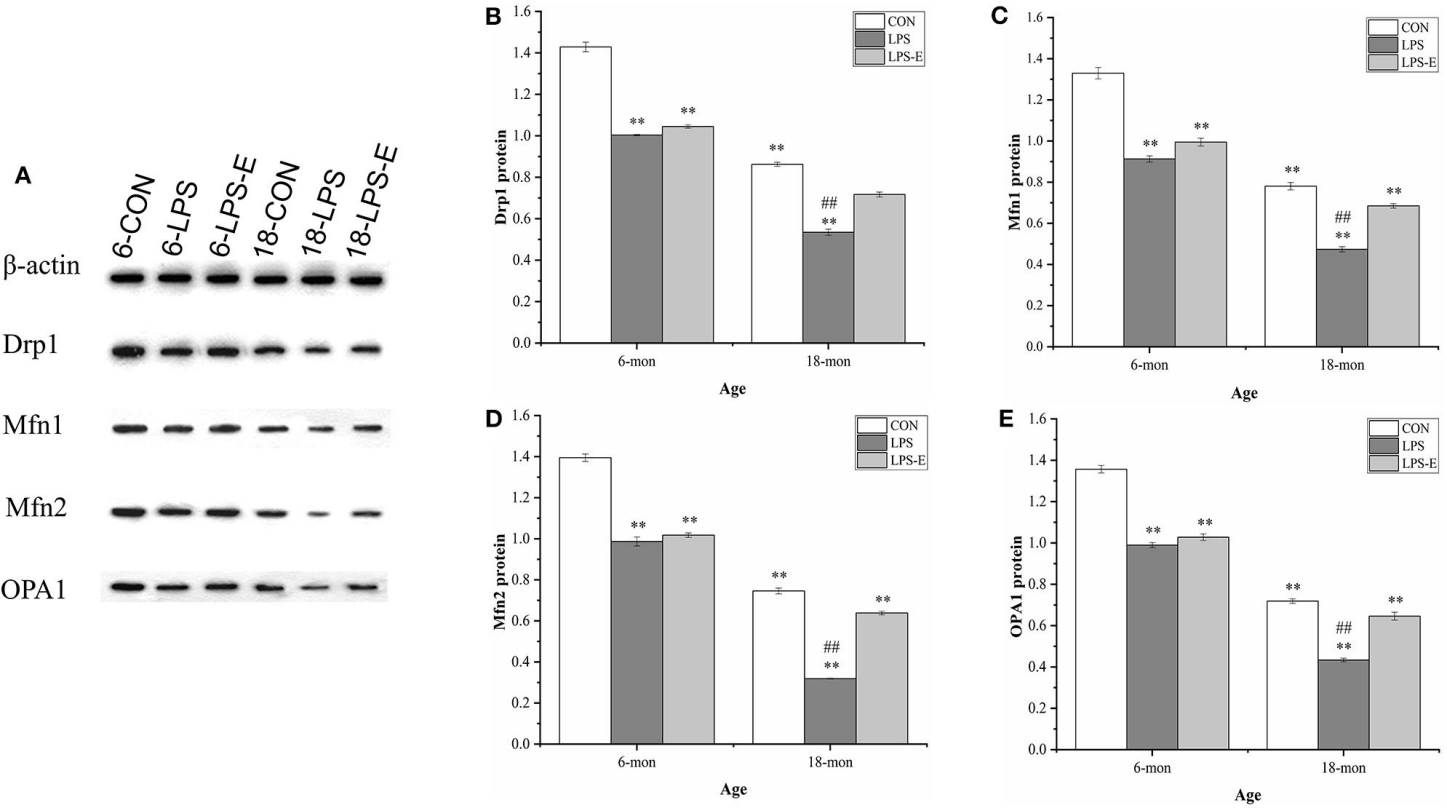
Protein expression of Drp1, Mfn1, Mfn2, and OPA1 in the hippocampus. **(A)** Representative gel of Drp1, Mfn1, Mfn2, and OPA1 protein expression in the different groups. **(B–E)** Differences in Drp1, Mfn1, Mfn2, and OPA1 protein levels in the different groups. *Significant differences compared with 6- or 18-month-old CON mice (**P < 0.01); ^#^Significant differences compared with 6- or 18-month-old LPS-E mice (^##^*P* < 0.01). 6: 6-month-old; 18: 18-month-old; CON, control group; LPS, lipopolysaccharide treatment group; LPS-E, LPS plus enriched environment treatment group.

As shown in Figures 3A–E, the 18-month-old mice from the CON group had significantly lower hippocampal protein levels of Drp1 (*t* = 23.237, *P* < 0.001), Mfn1 (*t* = 16.110, *P* < 0.001), Mfn2 (*t* = 28.402, *P* < 0.001), and OPA1 (*t* = 29.402, *P* < 0.001) than the 6-month-old mice from the same treatment group (CON). For the 6-month-old mice, the levels of Drp1 [*F*_(2,15)_ = 286.806, *P* < 0.001], Mfn1 [*F*_(2,15)_ = 103.411, *P* < 0.001], Mfn2 [*F*_(2,15)_ = 165.319, *P* < 0.001], and OPA1 [*F*_(2,15)_ = 163.493, *P* < 0.001] differed among the different treatment groups. Furthermore, the levels of all four proteins in the LPS group (*Ps* < 0.001) and those of Mfn1, Mfn2, and OPA1 in the LPS-E group (*Ps* < 0.05) were significantly downregulated when compared with the respective levels in the CON group; all four proteins showed similar expression levels in the LPS-E and LPS treatment groups (*Ps* > 0.05). In 18-month-old mice, the contents of Drp1 [*F*_(2,15)_ = 179.269, *P* < 0.001], Mfn1 [*F*_(2,15)_ = 113.760, *P* < 0.001], Mfn2 [*F*_(2,15)_ = 523.131, *P* < 0.001], and OPA1 [*F*_(2,15)_ = 112.420, *P* < 0.001] differed among the groups. Mice in both the LPS and LPS-E groups had significantly lower levels of the four proteins than mice in the CON group (*Ps* < 0.001); the levels of the four proteins were higher in the LPS-E-treated mice than in the LPS-treated mice (*Ps* < 0.001).

**Figure 4:**
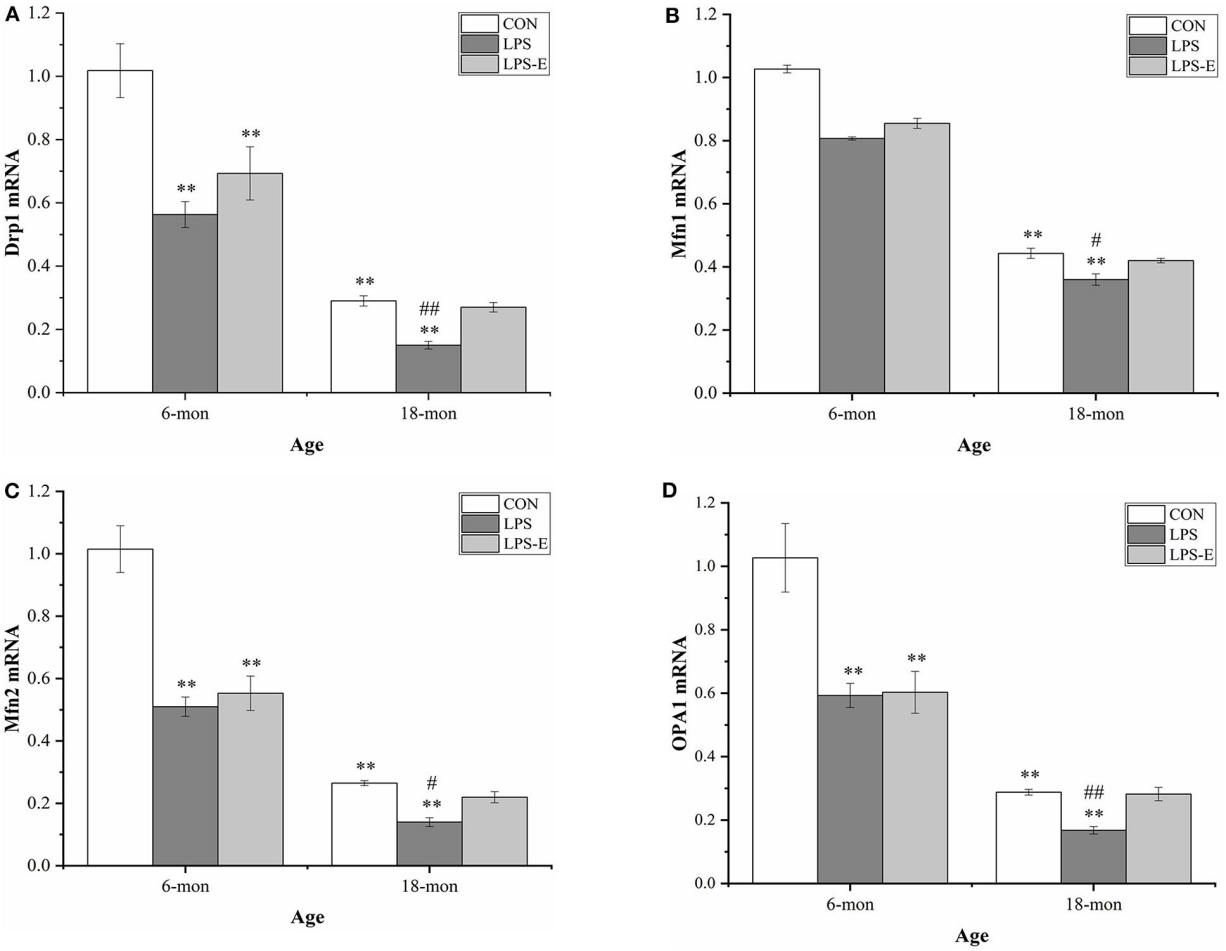
Relative mRNA levels of *Drp1, Mfn1, Mfn2*, and *Opa1* in the hippocampus. **(A–D)** Differences in *Drp1, Mfn1, Mfn2*, and *Opa1* in the different groups. *Significant differences compared with 6- or 18-month-old CON mice (**P < 0.01); ^#^Significant differences compared with 6- or 18-month-old LPS-E mice (^#^*P* < 0.05, ^##^*P* < 0.01). CON, control group; LPS, lipopolysaccharide treatment group; LPS-E, LPS plus enriched environment treatment group.

### Mitochondrial Biogenesis and Mitophagy

#### Messenger RNA Levels of *Pgc-1α, Pink1*, and Parkin

**Figure 5:**
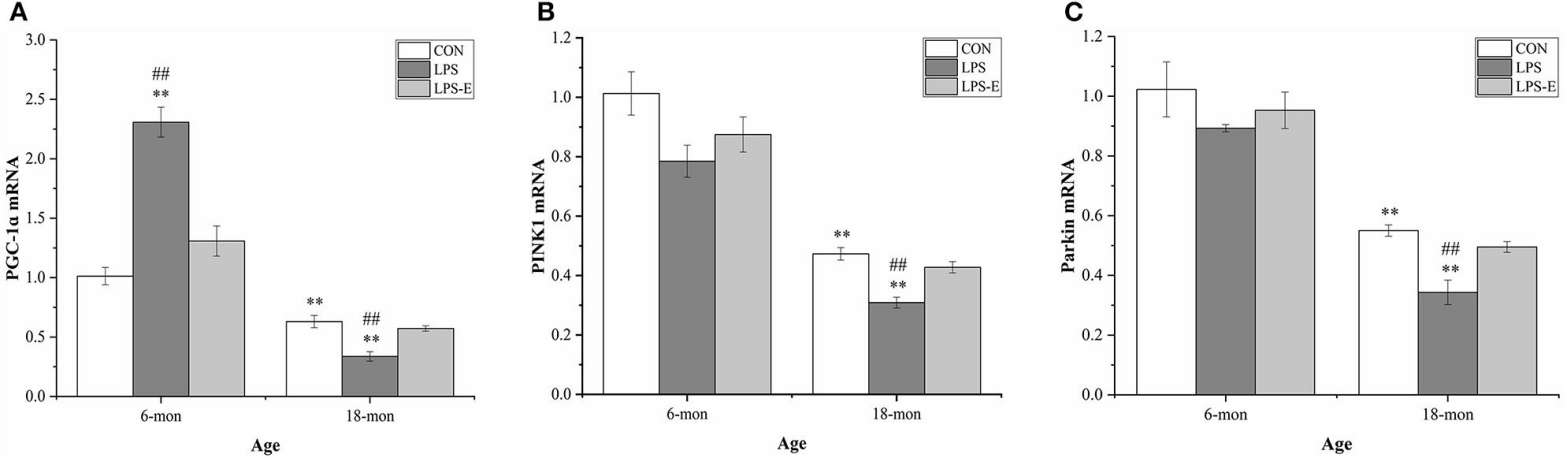
Relative mRNA levels of *Pgc-1*α, *Pink1*, and parkin in the hippocampus. **(A–C)** Differences in *Pgc-1*α, *Pink1*, and parkin mRNA levels in the different groups. *Denotes significant differences compared with 6- or 18-month-old CON mice (**P < 0.01); ^#^Denotes significant differences compared with 6- or 18-month-old LPS-E mice (^##^*P* < 0.01). CON, control group; LPS, lipopolysaccharide treatment group; LPS-E, LPS plus enriched environment treatment group.

Eighteen-month-old mice had significantly lower mRNA levels of *Pgc-1*α (*t* = 4.306, *P* = 0.002), *Pink1* (*t* = 23.237, *P* < 0.001), and parkin (*t* = 5.025, *P* = 0.003) than 6-month-old mice (Figures 5A–C). In 6-month-old mice, only *Pgc-1*α mRNA [*F*_(2,15)_ = 36.421, *P* < 0.001] levels differed among the groups, showing significantly higher expression in the LPS group than in the LPS-E and CON groups (*Ps* < 0.001), and marginally higher levels in the LPS-E group than in the CON group (*P* = 0.053). In 18-month-old mice, the levels of all the mRNAs [*F*_(2,15)_ = 15.411, *P* < 0.001 for *Pgc-1*α; *F*_(2,15)_ = 19.201, *P* < 0.001 for *Pink1*; and *F*_(2,15)_ = 14.465, *P* < 0.001 for parkin] differed significantly among the groups. Furthermore, the mRNA levels of the three genes were lower in the LPS treatment group than in the LPS-E and CON groups (*Ps* < 0.001); no differences were detected between the two latter groups (*Ps* > 0.05).

#### Protein Levels of PGC-1α, PINK1, and Parkin

**Figure 6:**
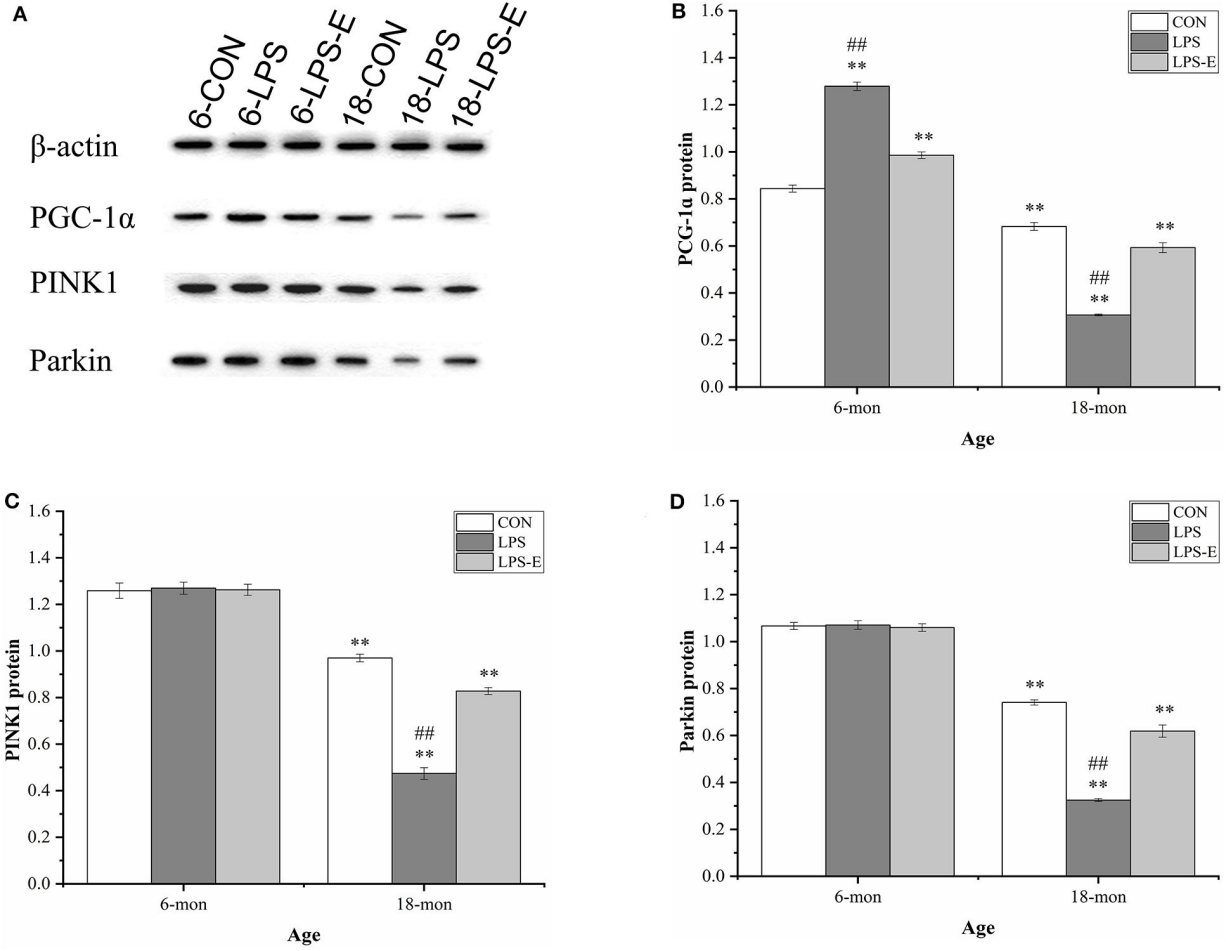
Protein expression of PGC-1α, PINK1, and parkin in the hippocampus. **(A)** Representative gel pattern of PGC-1α, PINK1, and parkin protein expression in the different groups. **(B–D)** Differences in Drp1, Mfn1, Mfn2, and OPA1 protein levels in the different groups. *Significant differences compared with 6- or 18-month-old CON mice (**P < 0.01); ^#^Significant differences compared with 6- or 18-month-old LPS-E mice (^##^*P* < 0.01). 6: 6-month-old; 18: 18-month-old; CON, control group; LPS, lipopolysaccharide treatment group; LPS-E, LPS plus enriched environment treatment group.

Similar to the mRNA levels, 18-month-old CON mice had lower protein levels of PGC-1α (*t* = 4.306, *P* < 0.001), PINK1 (*t* = 7.861, *P* < 0.001), and parkin (*t* = 17.404, *P* < 0.001) than 6-month-old CON mice (Figures 6A–D). In 6-month-old mice, only the PGC-1α protein level differed among the groups; PGC-1α protein levels were higher in the LPS and LPS-E treatment groups than in the CON group [*F*_(2,15)_ = 200.389, *P* < 0.001] and were also significantly higher in the LPS group than in the LPS-E group (*Ps* < 0.001). In 18-month-old mice, the protein levels of PGC-1α [*F*_(2,15)_ = 156.762, *P* < 0.001], PINK1 [*F*_(2,15)_ = 178.945, *P* < 0.001], and parkin [*F*_(2,15)_ = 162.081, *P* < 0.001] differed significantly among the groups. Slightly different from that seen for the mRNA levels, the concentrations of the three proteins were decreased in the LPS and LPS-E groups relative to the CON group (*Ps* < 0.001), as well as in the LPS group relative to the LPS-E group (*Ps* < 0.001).

### Correlations Between Morris Water Maze Performance and the Protein or Messenger RNA Levels of Mitochondrial Quality Control-Related Genes

For 6-month-old mice, the average learning-phase percent distance swam during the 7 days was negatively correlated with the hippocampal protein and mRNA levels of PGC-1α and Mfn2 in the LPS and LPS-E treatment groups (*Ps* < 0.05); the memory-phase percent distance swam or percent time spent swimming was positively correlated with the protein and mRNA levels of OPA1 in the LPS and LPS-E groups and with Mfn2 in the LPS-E group (*Ps* < 0.05).

For 18-month-old mice, the percent distance swam was negatively correlated with levels of all the mitochondrial-related proteins and mRNAs (biogenesis: PGC-1α; dynamics: Drp1, Mfn1, Mfn2, and OPA1; and mitophagy: PINK1 and parkin) in the three treatment groups (*Ps* < 0.05). The memory-phase percent distance swam and percent time spent swimming were positively correlated with the protein and mRNA levels of mitochondrial biogenesis (*Pgc-1*α), dynamics (*Drp1*), and mitophagy (*PINK1* and parkin)-related genes in each of the three groups (*Ps* < 0.05). In addition, the protein and mRNA levels of Mfn1, Mfn2, and OPA1 were positively correlated with both memory parameters in the CON and LPS-E groups (*Ps* < 0.05, Tables 2, 3); *Opa1* mRNA levels were also positively correlated with the percent distance swam in the LPS-E treatment group.

**Table 2:** Correlations between performances in the Morris water maze (MWM) and the levels of mitochondrial quality control-related proteins.

**Performance**	**Age**	**Group**	**PGC-1α**	**Drp1**	**Mfn1**	**Mfn2**	**OPA1**	**PINK1**	**Parkin**
Distance swam	6 months	CON	−0.134 (0.800)	0.046 (0.931)	−0.427 (0.399)	−0.311 (0.548)	−0.094 (0.860)	−0.006 (0.991)	−0.346 (0.501)
		LPS	−0.886 (0.019)^*^	−0.601 (0.207)	−0.104 (0.844)	−0.816 (0.048)^*^	−0.749 (0.087)	0.243 (0.643)	0.675 (0.141)
		LPS-E	−0.815 (0.048)_^*^_	−0.424 (0.403)	−0.566 (0.241)	−0.875 (0.023)^*^	−0.698 (0.123)	0.513 (0.298)	0.103 (0.846)
	18 months	CON	−0.850 (0.032)^*^	−0.866 (0.026)^*^	0.885 (0.019)^*^	−0.862 (0.027)^*^	−0.838 (0.037)^*^	−0.827 (0.042)^*^	−0.853 (0.031)^*^
		LPS	−0.837 (0.038)^*^	−0.850 (0.032)^*^	−0.811 (0.050)^*^	−0.885 (0.019)^*^	−0.893 (0.016)^*^	−0.917 (0.010)^**^	−0.915 (0.011)^*^
		LPS-E	−0.850 (0.032)^*^	−0.869 (0.025)^*^	−0.848 (0.033)^*^	−0.822 (0.045)^*^	−0.870 (0.024)^*^	−0.974 (0.001)^**^	−0.820 (0.046)^*^
Percent distance swam	6 months	CON	0.185 (0.252)	0.455 (0.364)	0.244 (0.641)	0.278 (0.594)	0.606 (0.202)	0.548 (0.260)	0.096 (0.976)
		LPS	−0.675 (0.141)	0.221 (0.674)	−0.370 (0.470)	0.640 (0.171)	0.813 (0.049)^*^	−0.148 (0.780)	−0.590 (0.218)
		LPS-E	0.669 (0.146)	0.708 (0.115)	0.584 (0.223)	0.838 (0.037)^*^	0.848 (0.033)^*^	−0.454 (0.365)	−0.264 (0.614)
	18 months	CON	0.968 (0.002)^*^	0.873 (0.023)^*^	0.876 (0.022)^*^	0.867 (0.025)^*^	0.9020 (0.014)^*^	0.982 (0.000)^**^	0.964 (0.002)^**^
		LPS	0.846 (0.034)^*^	0.862 (0.027)^*^	0.676 (0.140)	0.661 (0.153)	0.538 (0.271)	0.944 (0.005)^**^	0.924 (0.009)^**^
		LPS-E	0.926 (0.008)^**^	0.943 (0.005)^**^	0.688 (0.131)	0.737 (0.095)	0.790 (0.061)	0.911 (0.011)^*^	0.861 (0.028)^*^
Percent time spent swimming	6 months	CON	0.556 (0.252)	0.434 (0.390)	0.388 (0.447)	0.354 (0.491)	0.236 (0.652)	0.380 (0.458)	0.495 (0.318)
		LPS	−0.809 (0.051)	0.181 (0.732)	−0.136 (0.797)	0.769 (0.074)	0.837 (0.038)^*^	−0.041 (0.939)	−0.720 (0.106)
		LPS-E	0.769 (0.074)	0.196 (0.710)	0.654 (0.159)	0.967 (0.002)^**^	0.866 (0.026)^*^	−0.0135 (0.980)	−0.347 (0.500)
	18 months	CON	0.879 (0.021)^*^	0.973 (0.001)^**^	0.920 (0.009)^**^	0.880 (0.021)^*^	0.937 (0.006)^**^	0.870 (0.024)^*^	0.910 (0.012)^*^
		LPS	0.891 (0.017)^*^	0.872 (0.024)^*^	0.772 (0.072)	0.779 (0.068)	0.711 (0.113)	0.994 (0.000)^**^	0.985 (0.000)^**^
		LPS-E	0.908 (0.012)^*^	0.922 (0.009)^**^	0.876 (0.022)^*^	0.834 (0.039)^*^	0.865 (0.026)^*^	0.981 (0.001)^**^	0.887 (0.019)^*^

* Denotes significant correlation coefficients (*P < 0.05;

** *P < 0.01)*.

**Table 3:** Correlations between performances in the Morris water maze (MWM) and mRNA levels of mitochondrial quality control-related genes.

**Performance**	**Age**	**Group**	**Pgc-1α**	**Drp1**	**Mfn1**	**Mfn2**	**Opa1**	**Pink1**	**Parkin**
Distance swam	6 months	CON	−0.379 (0.459)	−0.076 (0.887)	−0.438 (0.385)	−0.305 (0.556)	−0.033 (0.951)	−0.326 (0.528)	−0.410 (0.420)
		LPS	−0.892 (0.017)^*^	−0.695 (0.125)	−0.741 (0.092)	−0.832 (0.040)^*^	−0.774 (0.071)	−0.347 (0.501)	−0.471 (0.346)
		LPS-E	−0.838 (0.037)^*^	−0.444 (0.378)	−0.217 (0.680)	−0.910 (0.012)^*^	−0.484 (0.331)	−0.309 (0.552)	−0.642 (0.169)
	18 months	CON	−0.814 (0.049)^*^	−0.947 (0.004)^**^	−0.813 (0.049)^*^	0.842 (0.036)^*^	−0.992 (0.000)^**^	−0.969 (0.001)^**^	−0.965 (0.002)^**^
		LPS	−0.906 (0.013)^*^	−0.858 (0.029)^*^	−0.892 (0.017)^*^	−0.841 (0.049)^*^	−0.774 (0.017)^*^	−0.982 (0.001)^**^	−0.820 (0.046)^*^
		LPS-E	−0.892 (0.017)^*^	−0.810 (0.019)^*^	−0.898 (0.015)^*^	−0.837 (0.038)^*^	−0.819 (0.017)^*^	−0.866 (0.019)^*^	−0.834 (0.039)^*^
Percent distance swam	6 months	CON	0.534 (0.275)	0.661 (0.153)	0.604 (0.204)	0.449 (0.372)	0.278 (0.594)	0.082 (0.877)	0.509 (0.303)
		LPS	−0.788 (0.062)	0.192 (0.715)	0.620 (0.189)	0.647 (0.165)	0.887 (0.018)^*^	0.435 (0.389)	0.720 (0.107)
		LPS-E	0.628 (0.181)	0.671 (0.145)	0.123 (0.817)	0.824 (0.044)^*^	0.568 (0.239)	0.620 (0.189)	0.786 (0.064)
	18 months	CON	0.911 (0.012)^*^	0.953 (0.003)^**^	0.842 (0.036)^*^	0.892 (0.017)^*^	0.824 (0.044)^*^	0.846 (0.034)^*^	0.855 (0.030)^*^
		LPS	0.941 (0.005)^*^	0.821 (0.045)^*^	0.517 (0.294)	0.738 (0.094)	0.780 (0.067)	0.811 (0.051)^**^	0.838 (0.037)^**^
		LPS-E	0.877 (0.022)^*^	0.817 (0.047)^*^	0.822 (0.045)^*^	0.847 (0.038)^*^	0.756 (0.082)	0.890 (0.017)^*^	0.898 (0.015)^*^
Percent time spent swimming	6 months	CON	0.706 (0.117)	0.359 (0.485)	0.743 (0.090)	0.671 (0.144)	0.489 (0.325)	0.589 (0.219)	0.340 (0.509)
		LPS	0.789 (0.062)	0.241 (0.646)	−0.770 (0.073)	0.750 (0.086)	0.827 (0.042)^*^	0.345 (0.503)	0.697 (0.124)
		LPS-E	0.514 (0.297)	0.737 (0.095)	0.530 (0.280)	0.893 (0.017)^**^	0.907 (0.012)^*^	0.572 (0.235)	0.791 (0.061)
	18 months	CON	0.929 (0.007)^**^	0.953 (0.003)^**^	0.840 (0.036)^*^	0.931 (0.007)^**^	0.901 (0.014)^*^	0.843 (0.035)^*^	0.832 (0.040)^*^
		LPS	0.937 (0.001)^**^	0.956 (0.003)^**^	0.705 (0.117)	0.794 (0.059)	0.796 (0.058)	0.961 (0.002)^**^	0.836 (0.038)^**^
		LPS-E	0.940 (0.005)^**^	0.876 (0.022)^*^	0.893 (0.016)^*^	0.881 (0.020)^*^	0.900 (0.014)^*^	0.906 (0.013)^*^	0.906 (0.013)^*^

* Denotes significant correlation coefficients (*P < 0.05;

** *P < 0.01)*.

## Discussion

### Aged Mice Exposed to a Long-Term Enriched Environment Exhibited Reduced Spatial Cognition Impairment Resulting From Lipopolysaccharide-Induced Gestational Inflammation

Memory impairment is the core symptom associated with several neurodegenerative diseases, such as AD. Impaired spatial memory that is associated with hippocampal structural and functional changes may be a preclinical sign of these diseases (Yassa et al., 2011). In the present study, in the MWM test, aged (18 months old) CON mice had longer swimming latency and swam longer distances in the place navigation task, as well as lower percent distance swam and percent time spent swimming in the target quadrant in the probe task, than young (6 months old) CON mice. These results suggested that learning and memory abilities had declined in the normal aged CD-1 mice. This was in accordance with previous results, including ours, which indicated that middle-aged (15 months old) CD-1 mice showed poorer spatial learning and memory performance in the six-radial arm water maze than the 6-month-old mice (Li et al., 2016).

Different types of stress have different effects on brain aging. Negative stresses, including poverty, low levels of education, and various diseases, increase the risk of AD (De Oliveira et al., 2014; Bae et al., 2015). In contrast, positive stresses, including marital satisfaction, good health, and abundant leisure activities, have been associated with cognitive preservation and a reduced incidence of cognitive impairment (De Oliveira et al., 2014; Liu et al., 2016; Sharifian et al., 2020). In rodents, a series of LPS exposure events may lead to accelerated age-related cognitive impairment (D'avila et al., 2018). Pregnant animals have increased sensitivity to LPS, which triggers maternal immune activation and leads to dysregulated gene expression in key brain regions in the mother, fetus, or both (Oskvig et al., 2012). This suggests that repeated exposure to LPS during pregnancy may accelerate brain senescence (Li et al., 2016). However, enriched housing conditions can delay age-related cognitive deficits (Doulames et al., 2014).

In the current study, we showed that, regardless of age (6 or 18 months), mice suffering from inflammation during gestation had poorer performance in the MWM test than control mice. These results suggested that exposure to LPS during pregnancy may impair spatial learning and memory in young mother mice and accelerate these processes in aged mice. Our results also indicated that LPS-E- and LPS-treated 6-month-old mice exhibited similar spatial learning and memory abilities. Hence, the ameliorative effect of an EE on the impaired spatial learning and memory resulting from exposure to gestational inflammation at ~3 months of age may not have had time to manifest in 6-month-old mice. However, LPS-E-treated 18-month-old mice had better spatial learning performance than those treated only with LPS and showed a similar performance to 18-month-old CON mice. These results indicate that shorter-term (<3 months) exposure to an EE did not alleviate the cognitive impairment resulting from inflammation during late pregnancy, whereas long-term exposure to an EE significantly delayed the progression of this cognitive decline in aged LPS-treated mice.

### Long-Term Exposure to an Enriched Environment Improved Dynamics in Hippocampi With Accelerated Aging

Mitochondrial structural integrity is essential for the normal function and longevity of this organelle (Luo et al., 2015). The changes in mitochondrial morphology that are modulated by mitochondrial fission and fusion (Saita et al., 2016) are a direct reflection of impaired mitochondrial structure and function (Muller-Rischart et al., 2013). Fission is mainly mediated by Drp1, whereas Mfn1/2 and OPA1 are important players in mitochondrial outer-membrane and inner-membrane fusion, respectively (Chen et al., 2011; Lee et al., 2017). Specifically, OPA1 is located in the inner mitochondrial membrane and maintains the integrity of mitochondrial cristae (Lee et al., 2017), where most oxidative phosphorylation occurs (Jimenez et al., 2014). In addition, fission can remove damaged mitochondrial components, whereas fusion can lead to the replacement of defective mitochondrial components from normal mitochondria, a process that is considered important for MQC (Leites and Morais, 2018). Hence, mitochondrial morphology and mitochondrial dynamics are interdependent for the maintenance of mitochondrial function.

In our study, Drp1, Mfn1, Mfn2, and OPA1 mRNA and protein levels were significantly lower in aged hippocampi than in young ones. These results suggested that mitochondrial dynamics undergo a gradual degeneration with senescence. In particular, the downregulation of OPA1 expression may be related to the destruction of mitochondrial cristae (Wu et al., 2019), resulting in the dysfunction of mitochondrial respiration in aged hippocampi (Silva K. A et al., 2019). Additionally, a decline in mitochondrial dynamics may be the cause of mitochondrial structural damage in normal aged mice (Lichvarova et al., 2018). Numerous studies have also shown that disrupted mitochondrial dynamics are associated with changes in mitochondrial morphology and function (Nie et al., 2019; Xu et al., 2019; Yan et al., 2019). This indicates that senescence is an important factor in changing mitochondrial structure and dynamics.

In addition to senescence, mitochondrial dynamics also are susceptible to adverse stresses (Hanqing et al., 2010; Kaufman et al., 2017; Meyer et al., 2018). Our findings suggested that an inflammatory insult in gestational mice at ~12 weeks of age would subsequently affect mitochondrial dynamics in the same mice 3 and 15 months after delivery. LPS-treated mice displayed the lowest levels of markers of mitochondrial dynamics in both age groups. Interestingly, long-term exposure to an EE could restore mitochondrial dynamics in aged mice. In our study, although an EE did not significantly improve the levels of markers of mitochondrial dynamics in 6-month-old mice, markers of mitochondrial dynamics were markedly increased in 18-month-old LPS-E-treated mice compared with LPS-treated mice of the same age. Because long-term exposure to an EE can reduce oxidative stress (Fernandez et al., 2004), we suspected that an EE might delay changes to mitochondrial dynamics in aged mice through antioxidant activity. Moreover, although 18-month-old LPS-E-treated mice also had lower levels of mitochondrial dynamics-associated proteins than CON mice, LPS-E-treated and CON mice displayed similar mRNA levels of mitochondrial dynamics-related genes, except for *Mfn*2. This difference may also be related to excess inflammation in the hippocampus, which may prevent some mRNAs from being transcribed and translated into protein. In brief, long-term exposure to an EE ameliorated the accelerated age-related changes in mitochondrial dynamics resulting from an inflammatory environment during gestation and may also have delayed the accelerated age-related cognitive decline.

### Long-Term Exposure to an Enriched Environment Alleviated the Accelerated Changes in Mitochondrial Biogenesis and Mitophagy in Aged Mice Resulting From Gestational Inflammation

To maintain mitochondrial homeostasis, cells must integrate two processes: removing damaged mitochondria (mitophagy) and replenishing healthy ones (mitochondrial biogenesis) (Leites and Morais, 2018). PGC-1α is a major regulator of mitochondrial biogenesis-associated gene transcription (Ventura-Clapier et al., 2008; Suliman and Piantadosi, 2014). PGC-1α is activated to meet cellular energy requirements under both physiological and pathological conditions, such as during exercise, starvation, or inflammation (Martin-Montalvo and De Cabo, 2013; Chen et al., 2018; Granata et al., 2018), which promotes mitochondrial biogenesis (Ventura-Clapier et al., 2008). Mitophagy plays a housekeeping role in maintaining a healthy mitochondrial pool through the selective removal of damaged mitochondria (Markaki et al., 2018). The PINK1/parkin pathway has a critical role in ubiquitin-dependent mitophagy (Held and Houtkooper, 2015). PINK1 can also affect fusion and fission processes by phosphorylating Mfn1/2 and Drp1, as well as other proteins located in the mitochondrial outer membrane (Lutz et al., 2009; Gegg et al., 2010; Poole et al., 2010). In the current study, we found that mitochondrial PGC-1α and PINK1/parkin levels were lower in 18-month-old mice than in 6-month-old mice. This suggested that biogenesis and mitophagy are reduced in aged mice, which might lead to the progressive accumulation of damaged mitochondria and age-related memory impairment (Lesnefsky et al., 2016). Consistent with our findings, accumulating evidence has indicated that defects in mitochondrial biogenesis and mitophagy are associated with aging and age-related diseases (Larocca et al., 2014; Picca et al., 2018; Shi et al., 2018).

For the effect of LPS, our results indicated that inflammation during gestation could lead to increased levels of PGC-1α in the hippocampi of the mice at 6 months of age, whereas at 18 months of age, the levels of PGC-1α would be reduced. Moreover, gestational inflammation did not affect the levels of PINK1/parkin at 6 months of age; however, the levels of both were significantly reduced at 18 months. Hence, LPS treatment exerted age-dependent effects on the patterns of mitochondrial biogenesis and mitophagy. Exposure to LPS during pregnancy can cause recent and remote effects on brain dysfunction. The former refers to inflammatory responses triggered by LPS (Hunter et al., 2007), whereas the latter refers to epigenetic modifications, including acetylation and methylation, in related genes in the mother (Chiariotti et al., 2016; Li et al., 2016). A recent study found that elderly people exhibiting high levels of CpG methylation in MQC-related genes are more prone to disability (D'aquila et al., 2019). In our study, we speculated that changes in mitochondrial biogenesis and mitophagy may be related to early inflammatory responses and later epigenetic modifications. Besides, an increase in mitochondrial biogenesis in young mice may be a compensatory mechanism for increasing the number of healthy mitochondria (Hunter et al., 2007). Meanwhile, mitophagy levels did not change significantly in young mice under the different treatments. This may have been due either to the recovery or elimination of damaged mitochondria through other MQC-related mechanisms, such as mitochondrial biogenesis and dynamics, in the hippocampi of young mice, or to the small sample size.

For the effect of an EE, our results showed that, at 6 months of age, LPS-E-treated mice had decreased levels of PGC-1α and similar levels of PINK1/parkin in the hippocampus compared with LPS-treated mice; however, the levels of all three proteins were increased at 18 months of age and were more similar to the levels seen in the CON mice. Although aged LPS-E mice had lower PINK1/parkin protein levels than aged CON mice, the mRNA levels were similar between both groups. These results indicated that long-term exposure to an EE could not only correct the change in mitochondrial biogenesis induced by an inflammatory insult during pregnancy but could also promote the expression of genes in the PINK1/parkin pathway at both the mRNA and protein levels, suggestive of an increase in mitophagy activity, especially in aged mice. Exposure to an EE is a simple and effective means of delaying brain aging (Birch and Kelly, 2019). In addition to its antioxidant effect, an EE can also alter the expression of important genes involved in brain aging through modifications in acetylation or DNA methylation patterns (Woldemichael et al., 2014). We speculate that an EE may also improve mitochondrial biogenesis and mitophagy through these effects; however, the specific underlying mechanisms need further investigation.

### Changes in Mitochondrial Quality Control Are Associated With Cognitive Abilities in the Different Age and Treatment Groups

Mitochondria are critical for neuronal energy metabolism, calcium regulation, myelination, and neuronal and glial apoptosis (Princz et al., 2018). Therefore, mitochondrial decay may be central to neuronal damage and cognitive decline (Lai et al., 2017; Cheng and Bai, 2018). To maintain mitochondrial function, various MQC mechanisms are integrated, including mitochondrial biogenesis, dynamics, and mitophagy, processes that coordinate to restore the normal mitochondrial pool (Fischer et al., 2012). Emerging evidence has indicated that the efficiency of MQC mechanisms declines with age (Larocca et al., 2014; Picca et al., 2018). Moreover, perturbing any one of these pathways in the brain can result in mitochondrial dysfunction and is sufficient to accelerate brain aging, thereby contributing to the development of neurodegenerative diseases (Chandra et al., 2019). For instance, a disrupted mitochondrial dynamics network and reduced levels of mitophagy are key early events in AD (Martin-Maestro et al., 2017). MQC mechanism malfunctioning may also be one cause of accelerated age-related cognitive impairment and changes in hippocampal neurobiochemical processes caused by LPS exposure during pregnancy in our previous study (Li et al., 2016). However, several natural compounds and drugs, as well as lifestyle changes, can ameliorate mitochondrial dysfunction and protect against age-associated pathological conditions through the regulation of mitochondrial dynamics and the induction of mitochondrial biogenesis and mitophagy (Suliman and Piantadosi, 2016). Consequently, MQC may be a potentially useful therapeutic target for improving age-related cognitive impairment.

In the current study, the results showed that spatial learning and memory abilities significantly decreased in parallel with MQC-related markers in 18-month-old mice. This suggests that the decline in hippocampal MQC is closely related to spatial learning and memory impairment. However, there were different patterns of correlation between MQC-related markers and MWM test performance in the two age groups under different treatments.

For normal control mice, all the earlier mentioned MQC markers were negatively correlated with the learning-phase distance swam and positively correlated with the memory-phase percent distance swam/percent time spent swimming in the 18-month-old mice. These results indicated that dysregulated MQC is associated with learning and memory impairment in normal aged mice.

For the LPS-treated mice, a negative correlation was found between the learning-phase distance swam and the protein and mRNA levels of PGC-1α and Mfn2 at 6 months of age, as well as all the MQC markers at 18 months of age. Similarly, a positive correlation was found between the percent distance swam/percent time spent swimming in the target quadrant and the protein and/or mRNA levels of Mfn2 and OPA1 in 6-month-old mice and the protein and mRNA levels of PGC-1α, Drp1, PINK1, and parkin in 18-month-old mice. These findings suggested that the impaired spatial learning and memory abilities may have been related to the changes in hippocampal mitochondrial biogenesis and dynamics in the younger LPS-treated mice and the reduced mitochondrial biogenesis, fission, and mitophagy in the older LPS-treated mice.

LPS- and LPS-E-treated mice showed similar correlation patterns; however, for the LPS-E-treated mice, positive correlations were also found between the memory-phase percent time spent swimming and the protein and mRNA levels of Mfn2 and OPA1 at 6 months of age and the protein and mRNA levels of Mfn1, Mfn2, and OPA1 at 18 months of age. These results suggested that the ameliorative effect of long-term exposure to an EE on the accelerated age-related spatial learning and memory impairment may have been attributable to increased mitochondrial fusion in the aged, PLS-treated mice.

### Summary

To the best of our knowledge, this study was the first to show that CD-1 mice exposed to LPS during pregnancy exhibit changed patterns of hippocampal MQC, including mitochondrial biogenesis, dynamics, and mitophagy, resulting in accelerated, age-related spatial learning and memory impairment. We also showed that long-term exposure to an EE might slow the acceleration of this age-related cognitive impairment by upregulating the mRNA and protein expression of MQC-related genes. This indicates that maintaining MQC homeostasis may be an important therapeutic target for the treatment of age-related cognitive impairment. Recent studies have shown that pharmacological or genetic manipulation of MQC processes can improve mitochondrial dysfunction (Suliman and Piantadosi, 2016); however, this is clinically challenging. In contrast, long-term exposure to an EE is a simple, safe, and non-invasive treatment that can improve cognitive behaviors and MQC mechanisms, as indicated in this study. Therefore, providing an EE or avoiding adverse stress is of great clinical significance in the prevention and treatment of age-related cognitive impairment. Nonetheless, the association between the protective effect of long-term exposure to an EE on the accelerated cognitive decline and MQC mechanisms could theoretically be a bystander effect, rather than causative. We did not investigate the treatment effects on mitochondria in the different regions of the whole hippocampus or MQC-related post-transcriptional translation, and further studies are needed to clarify the precise mechanism underlying MQC processes in different brain regions. In addition, in the future, we aim to investigate whether exposure to LPS during pregnancy has the same effect on offspring as it has on the mothers and whether the effect is dependent on epigenetic modifications in MQC-related genes.

## Data Availability Statement

The original contributions presented in the study are included in the article/supplementary materials, further inquiries can be directed to the corresponding author/s.

## Ethics Statement

The animal study was reviewed and approved by the Animal Ethics Committee of Anhui Medical University.

## Author Contributions

Z-QZ and Z-ZZ conceived and designed the study, performed a literature search and data acquisition, analyzed the data, and prepared the manuscript. Y-MZ, H-HG, and S-YS assisted in data acquisition, data analysis, and statistical analysis. PZ and G-HC reviewed the manuscript. All authors read and approved the content of the manuscript.

## Conflict of Interest

The authors declare that the research was conducted in the absence of any commercial or financial relationships that could be construed as a potential conflict of interest.
